# Fast 2D MRI acquisitions for motion correction in PET-MRI

**DOI:** 10.1186/2197-7364-1-S1-A58

**Published:** 2014-07-29

**Authors:** Michael Fieseler, Christopher Glielmi, Thomas Kösters, Lynn Frohwein, Fernado Boada, Xiaoyi Jiang, Klaus P Schäfers

**Affiliations:** European Institute for Molecular Imaging, University of Münster, Münster, Germany; Department of Computer Science, University of Münster, Münster, Germany; Center for Advanced Imaging Innovation and Research, NYU Langone Medical Center, New York, USA; Siemens Medical Solutions USA, New York, USA

We performed continuous, fast acquisitions of 2D MR slices covering the thorax under free breathing.

In present work, acquired 2D stacks are re-stored using a respiration signal. The usage of 2D slices is similar to the method described in [1]. The proposed method, however, does not include a navigator and acquisition times are shorter.

Dara were acquired from two patients on a Siemens Biograph mMR scanner (Siemens Healthcare, Erlangen, Germany) using a FLASH sequence, TE 1.38ms, TR 26ms, flip angle 12o, a 32-channel body-coil (acceleration factor of 8).

20 coronal slices were acquired with 3.9 x 6.25mm2 in-plane resolution (HF, LR), 128 x128 pixel, slice thickness 9mm, slice spacing 9mm, 26ms per 2D slice (total acquisition time 160s).

A respiratory signal was estimated from affine registrations of an area showing respiratory motion and subsequently used to sort the acquired 2D stacks into 8 respiratory phases.

**Figure 1:**
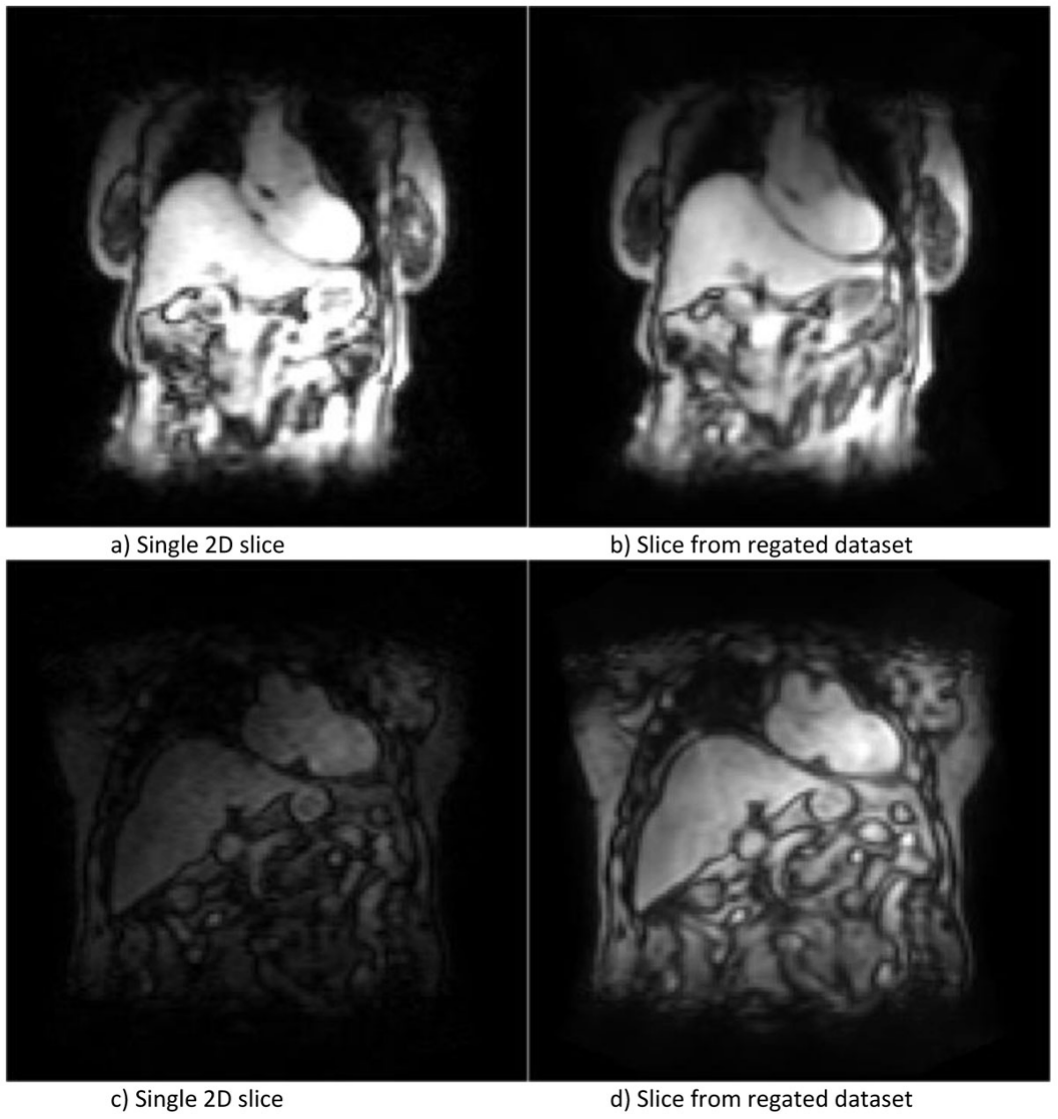
Datasets a) & c) show 2D slice of two acquired datasets. b) & d) show a slice from the re-gated, averaged datasets. Note the reduction of noise in the regated dataset.

Figure 1 shows input data and re-gated data. Noise is reduced by averaging of several 2D stacks. Additionally, cardiac motion is eliminated to a large extent, thus the generated dataset can be used for respiratory motion correction. Average correlation of 2D stacks assigned to a phase is 0.958 for dataset 1 (randomly selected gates: 0.933, SD 0.03). For dataset 2 the average correlation of 2D stacks assigned to a phase is 0.94 (randomly selected gates: 0.9, SD 0.036).

The results indicate that the proposed method is suitable for use in respiratory motion correction of PET data. In future work we will evaluate our approach on more datasets. Additionally, we will use motion estimates for each acquired 2D image stack to correct motion frame-wise.

